# An *in silico* pipeline to filter the *Toxoplasma gondii* proteome for proteins that could traffic to the host cell nucleus and influence host cell epigenetic regulation

**DOI:** 10.1590/0074-02760170471

**Published:** 2018-05-28

**Authors:** Genevieve Syn, Jenefer M Blackwell, Sarra E Jamieson, Richard W Francis

**Affiliations:** University of Western Australia, Telethon Kids Institute, Subiaco, Western Australia, Australia

**Keywords:** Toxoplasma gondii, nuclear localisation, epigenetics

## Abstract

*Toxoplasma gondii* uses epigenetic mechanisms to regulate both endogenous and host cell gene expression. To identify genes with putative epigenetic functions, we developed an *in silico* pipeline to interrogate the *T. gondii* proteome of 8313 proteins. Step 1 employs PredictNLS and NucPred to identify genes predicted to target eukaryotic nuclei. Step 2 uses GOLink to identify proteins of epigenetic function based on Gene Ontology terms. This resulted in 611 putative nuclear localised proteins with predicted epigenetic functions. Step 3 filtered for secretory proteins using SignalP, SecretomeP, and experimental data. This identified 57 of the 611 putative epigenetic proteins as likely to be secreted. The pipeline is freely available online, uses open access tools and software with user-friendly Perl scripts to automate and manage the results, and is readily adaptable to undertake any such *in silico* search for genes contributing to particular functions.

*Toxoplasma gondii* is a ubiquitous eukaryotic parasite associated with congenital or acquired ocular and/or brain lesions. It can influence the host’s transcriptome, including through modulation of the host epigenome ( Leng and Denkers 2009 , Leng et al. 2009 , Lang et al. 2012 ). The ability of infectious disease organisms to modulate the host epigenome is an area of increasing interest, which is likely to underpin development of novel therapeutics ( Vanagas et al. 2012 , Syn et al. 2016 ). Experimental studies of *T. gondii* have demonstrated the presence of epigenetic machinery, including histone ( Hettmann and Soldati 1999 ) and lysine ( Wang et al. 2014 ) acetyltransferases, histone deacetylases ( Vanagas et al. 2012 ), SET domain-containing histone lysine methyltransferases ( Sautel et al. 2007 ), SWI/SNF2 proteins capable of influencing gene expression through ATP-dependent nucleosome remodelling ( Sullivan Jr et al. 2003 ), and two predicted cytosine-5 DNA methyltransferase 2 (DNMT2) homologues ( Wei et al. 2017 ).

Availability of pathogen genome sequences provides the opportunity to undertake a more global approach to identify proteins mediating epigenetic effects, particularly in the context of increasing public domain data and tools that facilitate filtering approaches to identify all genes involved in specific molecular pathways. Here we develop an *in silico* pipeline to first identify all proteins in the *T. gondii* genome that can both target to eukaryotic cell nuclei and have putative epigenetic functions, and secondly to filter for those which also have secretory signals that could allow them to exit the parasite and affect the host epigenome directly. The pipeline is freely available (http://bioinformatics.childhealthresearch.org.au/software/nuc_loc/), uses open access tools and software with user-friendly Perl scripts to automate and manage the results, and is readily adaptable to undertake any *in silico* search for genes contributing to specific functions.

The source data used here was ToxoDB (http://toxodb.org) ( Gajria et al. 2007 ), which incorporates sequence and annotation from GenBank for the three clonal lineages of *T. gondii*: GT1 (Type I), ME49 (Type II) and VEG (Type III). We exported the *T. gondii* (ME49 strain) proteome of 8318 proteins. The filtering rationale ( Figure ) aimed to find all proteins predicted to: (i) go to a eukaryotic nucleus; (ii) have an epigenetic function; and (iii) be secreted from the parasite into the host cell. The pipeline and filtering parameters summarised below are described in more detail in the [ Supplementary data IV . Table I summarises the protein numbers from each step of the *in silico* pipeline. Supplementary data I contains both the raw and summary results arising from running all 8318 *T. gondii* encoded proteins through all *in silico* tools.

**Figure:**
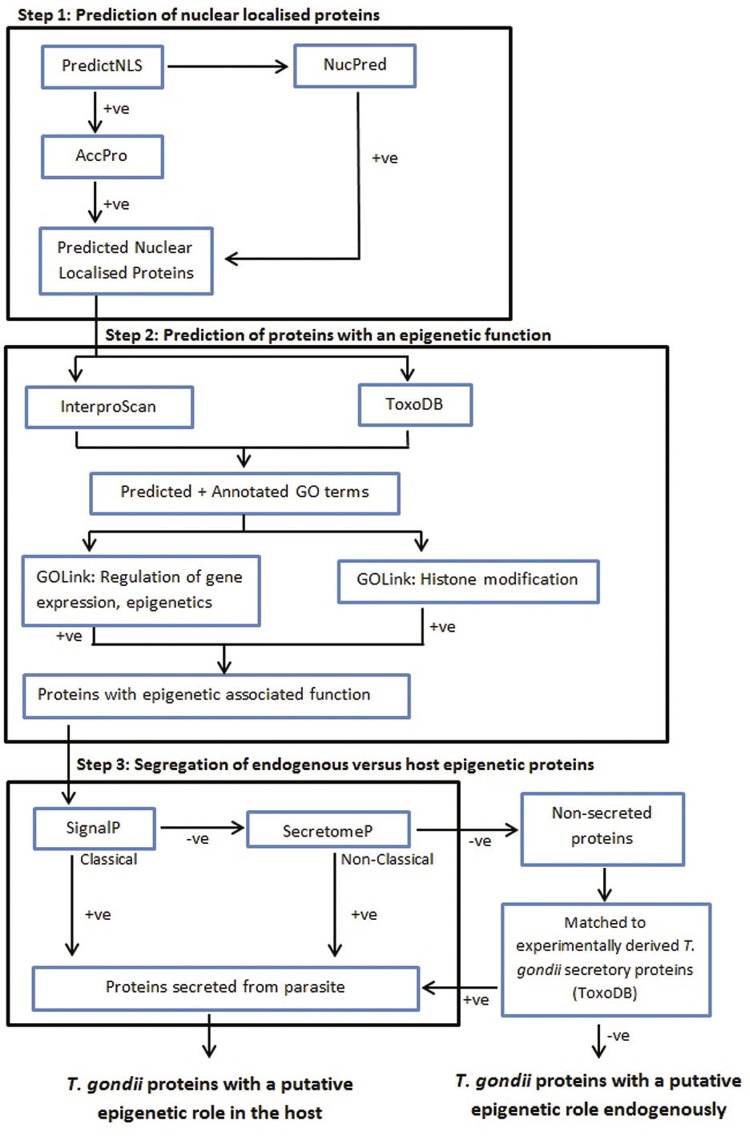
Bioinformatics pipeline outlining the *in silico* tools and manual methods used to predict parasite proteins with endogenous and exogenous epigenetic function. Step 1: predicts proteins localised to the nucleus; Step 2: predicts proteins with potential epigenetic function; Step 3: applies a filter to determine potential for epigenetic function in the host versus endogenous epigenetic function.

**TABLE I:** Summary of proteins filtered through the *in silico* pipeline. The pipeline was designed to identify a set parasite encoded candidates in the putative secretome that could potentially target the host or parasite nucleus and have domains consistent with an epigenetic function

	Proteins retained ^*^	Overall percentage of the proteome
Initial *Toxoplasma gondii* proteome	8318	100%
Step 1: prediction of nuclear localised proteins	3408	41%
Step 2: proteins from Step 1 with a predicted epigenetic function	611	7.4%
Step 3a: proteins from Step 2 with a predicted epigenetic function in host	57	0.7%
Step 3b: proteins not included in Step 3a: predicted epigenetic function in parasite	554	6.7%

*: protein numbers retained and percent of the entire proteome at each step of the *in silico* pipeline.

To predict proteins with the potential to localise to eukaryotic cell nuclei, we first used PredictNLS (version 1.3) ( Cokol et al. 2000 ) combined with AccPro ( Magnan and Baldi 2014 ) to identify proteins containing an exposed putative nuclear localisation signal (NLS), and calculated the overall percentage of exposed residues for each NLS motif. Some proteins, whilst not themselves containing an NLS, gain access to the nucleus when bound to an NLS-containing protein. Proteins may also contain novel NLSs. To capture proteins such as these we used NucPred (version 1.1), a tool that predicts whether or not proteins spend time in the nucleus ( Brameier et al. 2007 ). Of the 8318 *T. gondii* encoded proteins extracted from ToxoDB, 3408 were predicted to have the potential to localise to a eukaryotic nucleus. This included 1399 proteins that contain a putative functional exposed NLS motif, as predicted by PredictNLS/ACCpro, and 3201 proteins predicted to spend some time in the nucleus by NucPred. There was an overlap of 1192 proteins. The 2009 proteins predicted to be nuclear localised by NucPred alone could contain as yet unidentified NLSs or be chaperoned into the nucleus.

To predict proteins with an epigenetic function we used InterProScan Version 5 ( Zdobnov and Apweiler 2001 ), a tool that allows users to query protein sequences against the InterPro database ( Hunter et al. 2012 ). InterProScan predicts structural motifs and domains and provides any available associated Gene Ontology (GO) terms. We also exported a list of manually annotated and predicted GO terms from ToxoDB (version 8.0) for all *T. gondii* proteins. To allow inference of epigenetic function from GO terms across the orthogonal sub-ontologies (“Cellular Component”, “Molecular Function” and “Biological Process”) we used GOLink ( Francis 2013 ), a tool that collates terms across all sub-ontologies that co-occur with a term of interest across the entire GO database. We used GOLink to compile “terms lists” linked to the query terms “regulation of gene expression, epigenetic’’ (GO:0040029) and “histone modification” (GO:0016570). We removed the generic GO term “Protein binding” from both lists to reduce false positives. The two GOLink terms lists used in this process can be found at Supplementary data II . All associated GO terms for each protein were then matched to the top 5% of terms from each of the generated GOLink “terms lists” to determine those parasite proteins predicted to have an epigenetic function. The terms within the 95th percentile were selected as they represent the terms most confidently associated with the initial query terms. Of the 3408 proteins with a prediction of nuclear localisation, 1487 had associated GO terms. The GOLink terms lists ( Supplementary data II ) obtained for “regulation of gene expression, epigenetic” and “histone modification” contained 140 and 168 terms respectively (representing the top 5% of all candidate terms returned by GOLink) with an overlap of 68 terms, leaving 240 unique terms that were used in the matching process. Filtering of these 1487 proteins using our GOLink terms lists resulted in 611 proteins ( Supplementary data III ) that were deemed to be associated with epigenetic functions.

To identify *T. gondii* encoded proteins that may play a role in modulating the host cell epigenome we used SignalP (version 4.1) to predict proteins with an N-terminal signal peptide (SP) for secretion via the classical secretory pathway. We used the Eukaryote organism groups for our predictions and default D-cutoff values of 0.45 for the SignalP-noTM networks and 0.50 for SignalP-TM networks. Any proteins with a D-score exceeding the relevant cutoff were deemed as having a signal peptide and were included in a putative parasite secretome. As Eukaryotic proteins can be secreted without a classical N-terminal signal peptide, we also used SecretomeP (version 2.0) ( Bendtsen et al. 2004 ) to predict *T. gondii* proteins likely to be secreted via a non-classical secretory pathway. This tool is used in conjunction with SignalP: any protein predicted by both SecretomeP and SignalP is likely to be classically secreted, prediction by SecretomeP only suggests a non-classical mechanism. Proteins with a SecretomeP NN-score >0.9 ( Bendtsen et al. 2004 ) were included in the putative parasite secretome. Of the 611 *T. gondii* proteins with a predicted epigenetic function, 23 were predicted to be secreted via the classical pathway and 1 via the non-classical pathway.

We noted that GRA10 (TGME49_268900) previously shown ( Ahn et al. 2007 ) to be secreted from *T. gondii* into the host cell and targeted to the host cell nucleus was not predicted to be secreted by either SignalP or SecretomeP. This suggests alternative mechanisms for *T. gondii* proteins to be secreted, for example, the recently reported ( Coffey et al. 2015 ) aspartyl pathway. To address this, the 587 epigenetic candidates with no prediction of secretion by either SignalP or SecretomeP were further screened to determine if any had been proposed to be secreted through experimental studies, in particular the published experimental secretome ( Zhou et al. 2005 ). This yielded an additional 33 epigenetic candidates that were deemed secreted. One caveat in using this experimental secretome data was the observation ( Zhou et al. 2005 ) that a number of cytosolic proteins identified in the secretome could be contaminants due to inadvertent lysis of the parasites during sample preparation. However, since there was no way of distinguishing such false positives from true secreted proteins that might carry novel, possibly *T. gondii* -specific ( Coffey et al. 2015 ), secretory signals we retained the full list of proteins from the experimental secretome in our gene list. This included retaining all proteins that had at least one peptide from the mass spectrometry analysis that mapped to the *T. gondii* database, since there was high probability given the experimental conditions that all peptides identified with a high confidence spectral match to the *T. gondii* genome were of parasite origin. A second caveat was that the experimental secretome contained very few dense granule and no rhoptry proteins, most likely due to the use of 1% ethanol to stimulate parasite secretion ( Zhou et al. 2005 ). In view of this, the putative secretome list was extended through a search of ToxoDB using keywords such as “Rhoptry”, “Dense Granule” and “Microneme”.

Overall, 57 proteins were predicted to be secreted and have an epigenetic function ( Table II ). These proteins represent 0.7% of the total *T. gondii* proteome and are considered candidates for translocation to the host cell nucleus during infection with a potential role in the manipulation of the host epigenome. The 554 epigenetic candidates not predicted to be secreted were considered to have an endogenous role in the epigenetic regulation of *T. gondii.*

**TABLE II:** Proteins from our pipeline with a potential epigenetic function in modulating the host epigenome. The list is annotated for proteins identified from the *in silico* secretome, the experimental secretome data, or both

ToxoDB ID ^*a*^	Protein description ^*a*^
*In silico* secretome	
TGME49_207690	PDCD5
TGME49_213310	hypothetical protein
TGME49_239440	protein kinase (incomplete catalytic triad)
TGME49_241870	tRNA ligase class I (E and Q), catalytic domain-containing protein
TGME49_243280	Met-10+ like-protein
TGME49_245660	hypothetical protein
TGME49_257010	sporozoite developmental protein
TGME49_271625	serine--tRNA ligase
TGME49_277030	isoleucyl-tRNA synthetase, putative
TGME49_281675	hypothetical protein
TGME49_284010	5’-3’ exonuclease, N-terminal resolvase family domain-containing protein
TGME49_295050	tRNA ligase class II core domain (G, H, P, S and T) domain-containing protein
TGME49_299810	cysteine-tRNA synthetase (CysRS)
TGME49_305920	endonuclease III family 1 protein
TGME49_312370	RNA pseudouridine synthase superfamily protein
TGME49_312520	tRNA dimethylallyltransferase
TGME49_313120	DNA-directed RNA polymerase, alpha subunit
Experimental secretome	
TGME49_202490	AP2 domain transcription factor AP2VIIa-7
TGME49_206510	toxolysin TLN4
TGME49_207080	histone lysine acetyltransferase MYST-B
TGME49_210310	hypothetical protein
TGME49_210360	DEAD (Asp-Glu-Ala-Asp) box polypeptide 41 family protein
TGME49_219600	hypothetical protein
TGME49_223880	zinc finger, C3HC4 type (RING finger) domain-containing protein
TGME49_224480	cell-cycle-associated protein kinase CLK, putative
TGME49_226510	Sec23/Sec24 trunk domain-containing protein
TGME49_228120	hypothetical protein
TGME49_231170	RecF/RecN/SMC N terminal domain-containing protein
TGME49_239420	protein kinase
TGME49_240090	rhoptry kinase family protein ROP34, putative
TGME49_246060	polymerase (RNA) mitochondrial (DNA directed) POLRMT
TGME49_246760	hypothetical protein
TGME49_252500	polo kinase
TGME49_253750	PLU-1 family protein
TGME49_253890	peptidase M16 inactive domain-containing protein
TGME49_267030	ribonuclease type III Dicer
TGME49_268900	dense granular protein GRA10
TGME49_269885	rhoptry metalloprotease toxolysin TLN1
TGME49_271290	hypothetical protein
TGME49_271740	hypothetical protein
TGME49_278440	SWI2/SNF2 Brahma-like putative
TGME49_285895	AP2 domain transcription factor AP2V-2
TGME49_289330	ubiquitin carboxyl-terminal hydrolase family 2 protein
TGME49_292055	calcium dependent protein kinase CDPK8
TGME49_292235	hypothetical protein
TGME49_294840	zinc finger (CCCH type) motif-containing protein
TGME49_305750	nucleolar gtp-binding protein 2, putative
TGME49_306660	RNA pseudouridine synthase superfamily protein
TGME49_312830	hypothetical protein
TGME49_313330	rhoptry kinase family protein ROP27
Both *in silico* and experimental secretome	
TGME49_201130	rhoptry kinase family protein ROP33
TGME49_207610	rhoptry kinase family protein ROP36 (incomplete catalytic triad)
TGME49_221330	DNA gyrase/topoisomerase IV, A subunit domain-containing protein
TGME49_229630	eIF2 kinase IF2K-A (incomplete catalytic triad)
TGME49_262730	rhoptry protein ROP16
TGME49_294560	rhoptry kinase family protein ROP37 (incomplete catalytic triad)
TGME49_309110	tRNA methyl transferase

*a*: retrieved from ToxoDB (Version 8.0).

To date, six *T. gondii* secreted proteins have been experimentally shown to target the host cell nucleus. Rhoptry kinase family proteins 16 (ROP16) ( Saeij et al. 2007 ) and 47 (ROP47) ( Camejo et al. 2014 ), protein phosphatase 2C (PP2C-hn) ( Gilbert et al. 2007 ), and dense granular proteins 10 (GRA10) ( Ahn et al. 2007 ), 16 (GRA16) ( Bougdour et al. 2013 ) and 24 (GRA24) ( Braun et al. 2013 ). In addition, we report three proteins that have domains associated with histone modifications: TGME49_207080 (TgMYST-B), which contains a histone acetyltransferase domain; TGME49_202490 containing a histone methylation SET domain; and TGME49_210310 which is predicted to have a histone methylation DOT1 domain. It was previously thought that *T. gondii* did not possess any DOT1 domain histone methyltransferases ( Sullivan Jr and Hakimi 2006 ) but prediction of functional domains using InterProScan now highlights TGME49_210310 as containing a DOT1 domain. The orthologue of TGME49_210310 in the VEG strain of *T. gondii* (TGVEG_210310) is also annotated as having a DOT1 domain in ToxoDB, adding confidence to our prediction of this DOT1 domain in TGME49_210310. Our *in silico* analysis also identified one protein involved in nucleosome remodelling, TGME49_278440, which contains a SWI/SNF2 related bromodomain. SWI/SNF members are capable of ATP-dependent destabilisation of histone-DNA interactions. Finally, we report TGME49_228120, currently annotated in ToxoDB as having a “replication foci targeting sequence” of the type uniquely found in the N-terminal region of DNMT1 molecules ( Subramaniam et al. 2014 ). *T. gondii* lacks detectable methylcytosine in its DNA ( Gissot et al. 2008 ), and has no full-length DNMT1 homologues. The C-terminal 500 amino acid catalytic portion of DNMTs are conserved between C5 DNMTs of eukaryotes and prokaryotic organisms, and harbour the active centre of the enzyme containing amino acid motifs characteristic of the cytosine-C5-methyltransferase. The N-terminal region of DNMT1 molecules carries 3 sequences, including the replication foci targeting sequence, that increase the precision in copying methylation patterns after DNA replication. As this replication foci targeting sequence is unique to DNMT1 molecules, this suggests that TGME49_228120 may represent the remnants of a *T. gondii* DNMT1 gene.

Overall this *in silico* analysis has identified several highly interesting candidates for ongoing functional investigations into how *T. gondii* manipulates host cell processes, specifically via the disruption or modulation of the host epigenome. Recently, it has been proposed that distribution of secretory pathogenesis determinants (genes encoding secretory proteins found in MIC, DG, ROP and SRS superfamilies) within the *T. gondii* genome is strain-specific ( Lorenzi et al. 2016 ). Since we have only analysed the ME49 (Type II) proteome, the proteomes of other *T. gondii* strains can be analysed using our freely available pipeline to identify strain-specific proteins that may affect host epigenetics. With the discovery of compounds which act against parasites by inhibiting histone deacetylase ( Vanagas et al. 2012 ), there is increasing interest in identifying the parasite proteins involved in epigenetic regulation, both endogenously and within the host. These proteins represent potential novel drug targets for suppression or treatment of toxoplasmosis.
