# Supplementary material for: An in silico pipeline to filter the Toxoplasma gondii proteome for proteins that could traffic to the host cell nucleus and influence host cell epigenetic regulation
Source: Mem Inst Oswaldo Cruz. 2018 May 28;113(6):e170471. doi: 10.1590/0074-02760170471 (PMC5963570; doi:10.1590/0074-02760170471)
Supplement: Supplementary file 2 [file 0074-0276-mioc-113-6-e170471-Suppl01.pdf]

METHODS

ToxoDB (Gajria et al. 2007) is the primary genome resource for *Toxoplasma gondii* containing both genomic and functional data. It incorporates sequence and annotation from GenBank for the three clonal lineages of *T. gondii*: GT1 (Type I), ME49 (Type II) and VEG (Type III) (Gajria et al. 2007). Using ToxoDB (Version 8.0), we extracted the protein sequences of the entire *T. gondii* proteome. This was done by first selecting "Gene type" under the heading "Gene Attributes". The search parameters were then defined as Organism = "*Toxoplasma gondii* ME49" and Gene type = "protein coding" All proteins was then downloaded in FASTA format after selecting "Download 8313 Genes". In addition, the list of Gene Ontology (GO) terms associated with all *T. gondii* proteins was generated in ToxoDB. Under the formatting option "Text: choose from columns and tables", GO terms manually annotated by other users or predicted using the InterPro database (version 39.0) were selected for download in summary text files.

We also created an initial list of parasite secreted proteins by searching through "Mass Spec. Evidence" under the heading "Protein Expression" and exporting data from "Tachyzoite Secretome (RH)" (Zhou et al. 2005). This generated a list of proteins from the RH strain of *T. gondii* experimentally shown to be secreted from the parasite using a variety of methods. Notably this list contained very few dense granule and no rhoptry proteins. This is most likely due to the fact that 1% ethanol was used to stimulate parasite secretion. Ethanol is known to increase Ca^{2+} levels (Carruthers et al. 1999), which in turn stimulates microneme secretion but does not have much effect on rhoptries or dense granules (Carruthers et al. 1999). In view of this, the protein list was extended through a search of ToxoDB using keywords such as "Rhoptry", "Dense Granules" and "Microneme" to find additional proteins of the secretory organelles. All data were exported in FASTA format and summary text files.

Firstly PredictNLS (version 1.3) combined with AccPro was used to identify proteins containing an exposed putative NLS. NLS motifs are identified by pattern matching against a predefined set of NLS regular expressions (RE). These expressions are based on a set of 91 experimentally validated NLS sequences that have been expanded through *in silico* mutagenesis to create a reference set of 214 potential NLS REs that match 43% of all known nuclear proteins but no non-nuclear proteins (Cokol et al. 2000). Whilst the exact location of an NLS motif within the amino acid sequence can be variable (Dingwall et al. 1991) it must be surface exposed for it to be recognised by the nuclear transport machinery and thus be functional. This property can be assessed by looking at the solvent accessibility (SA) of all protein sequences deemed to contain one or more NLS motif using ACCpro (version 4.1), part of the SCRATCH protein structure prediction server (Pollastri et al. 2002, Cheng et al. 2005). It applies a neural network trained on PSI-BLAST profiles of proteins from the Protein Data Bank (PDB) to accurately (77% at the 25% exposure threshold) predict a binary (exposed/buried) state category of solvent accessibility for each amino acid in a given protein sequence. ACCpro was run with default parameters, using BLAST data from NCBI's nr database dated June 2012. The Perl script we provide (http://bioinformatics.childhealthresearch.org.au/software/nuc_loc/) is able to parallelise the analysis process and specifically assess the exposed/buried state of each residue in the NLS motif. In the ACCpro output, each amino acid residue is classified as either "e", which predicts it is exposed, or "b", which predicts that it is buried. The overall percentage of exposed residues for each NLS motif was then calculated separately using this data. For bipartite NLS sequences (identified by a linker sequence of 10 or more amino acids), the SA status of the linker sequence amino acids was excluded from the calculation. NLS motifs with an exposure level of greater than 70% were deemed "exposed".

It is also known that some proteins, whilst not themselves containing a NLS, can gain access to the nucleus when bound to a protein that does contain a functional NLS (Tinland et al. 1992). Proteins may also contain novel NLSs. To capture proteins such as these we also used NucPred (version 1.1), a tool which predicts whether or not proteins spend time in the nucleus (Brameier et al. 2007). It applies a machine learning approach (genetic programming) using 100 sequence based predictors, comprising multiple REs derived from UniProt proteins whose nuclear localisation status is known. Query sequences are matched against each predictor's REs to determine if the sequence has a propensity to be nuclear or not. The proportion of predictors reporting a nuclear localisation forms the NucPred score. NucPred was used with default parameters and proteins having a NucPred score of ≥ 0.7 were selected as having spent some time in the nucleus. This NucPred score is reported to offer a specificity value of 0.81 and a sensitivity value of 0.44 in a prediction of a protein's nuclear localisation potential (http://www.sbc.su.se/~maccallr/nucpred/; Brameier et al. 2007). The NucPred score is reported to offer a specificity value of 0.81 and a sensitivity value of 0.44 in a prediction of a protein's nuclear localisation potential. Unlike PredictNLS, NucPred is not constrained to known NLS motifs giving it the potential to identify proteins that may contain novel NLSs.

InterProScan (Zdobnov and Apweiler 2001) allows users to query protein sequences against the InterPro database, (Hunter et al. 2012) a resource created to integrate multiple protein sequence classification databases. InterProScan predicts structural motifs and domains as provided by each integrated InterPro database, along with any available GO terms (Ashburner et al. 2000). The GO is a controlled vocabulary of terms designed to be used as annotation for genes and gene products. It is split into three sub-ontologies; "Cellular Component", "Molecular Function" and "Biological Process". By design there is no standard method to link between the sub-ontologies, although some techniques have been proposed (Mungall 2004, Bada and Hunter 2007, Mei et al. 2011, Chi and Nam 2012). To allow inference of epigenetic function from GO terms across all sub-ontologies we used GOLink (Francis 2013). This tool takes a parent GO term (such as GO:0005634 for nucleus) and extracts any gene annotated with that parent term or any of its child terms from the GO database (Ashburner et al. 2000). Next GOLink assesses each extracted

CC BY

gene's annotated GO terms, which are assigned to any of three separate, increasingly stringent, GOLink "terms lists" so long as the gene's annotation fulfils the list's specific criteria. Overall each list contains "co-occurring" terms from all GO sub-ontologies that fit the list criterion and can subsequently be confidently associated with the initial query term. We used GOLink to compile terms lists of "Cellular Component", "Molecular Function" and "Biological Process" terms linked to the "Biological Process" query terms "regulation of gene expression, epigenetic'' (GO:0040029) and "histone modification" (GO:0016570). It should be noted that the latter is not a child term of the former or vice versa. The generated terms were not restricted to any particular source database but were subject to filtering for terms annotated with the Inferred from Electronic Annotation (IEA) evidence code.

InterProScan (version 5) was used to search all submitted proteins for functional domains. Each protein's predicted domain-associated GO terms combined with any additional ToxoDB GO annotation were matched against the top 5% of terms from each of the GOLink "all list" terms lists to determine those proteins predicted to have an epigenetic function. Terms in the 95th percentile of the "terms list' were used as they represent those terms most confidently associated with the initial query terms. As recommended in the GOLink article, we removed the generic term "Protein binding" to reduce false positives prior to filtering. A full list of the GO terms included in this search can be found at Supplementary data II.

SignalP (version 4.1) was used to assess whether or not submitted *T. gondii* proteins were likely to possess an N-terminal signal peptide (SP) and be secreted from the parasite via the classical secretory pathway. SignalP uses two neural networks trained on high quality positive and negative datasets that are designed to distinguish between a true signal peptide and N-terminal transmembrane domains. This is achieved by assigning a score to each amino acid based on the likelihood that it belongs to either the SP (s-score) or to the mature protein (c-score). The y-score is then derived from the s-score and c-score to predict where the SP is cleaved and finally the d-score is derived from the y-score and s-score and used to better distinguish the presence of an SP and its cleavage site. We used the Eukaryote organism groups for our predictions and default D-cutoff values of 0.45 for the SignalP-noTM networks and 0.50 for SignalP-TM networks. Any proteins with a D-score exceeding the relevant cut-off were deemed as having a signal peptide and were included in the putative parasite secretome.

SecreteomeP was also used as a small number of proteins, such as fibroblast growth factor 2 (Nickel 2011) and Interleukin-1 (Rubartelli et al. 1990), are known to be secreted without a classical N-terminal signal peptide. In view of this we also used SecreteomeP (version 2.0) to predict whether any submitted *T. gondii* proteins were likely to be secreted in such a similar leaderless manner or via a non-classical secretory pathway (Bendtsen et al. 2004). SecretomeP predictions are based upon a neural network that was trained on positive data comprising known classically secreted protein sequences with their SP removed and a negative dataset containing proteins known to only localise to the cytoplasm or the nucleus. From these datasets a number of sequence-derived features were revealed to correlate with localisation such as number of atoms and number of positively charged residues. For each submitted query sequence these features are calculated and a score (NN-score) assigned indicating the likelihood the protein is secreted or not. NN-scores above 0.5 indicate possible secretion. This tool is designed to be used in conjunction with SignalP in that any protein predicted by SecretomeP to be secreted that also has an SP as predicted by SignalP is likely to be classically secreted. In the absence of an SP a prediction of secretion by SecretomeP suggests a potentially non-classical mechanism. Input sequences were split into groups of 100 sequences and manually submitted to the SecretomeP2 web server, selecting the mammalian prediction model. An in-house Perl script was used to merge the data with the main dataset. Those with an NN-score above 0.9 were considered as being secreted and these proteins were also included in the putative parasite secretome.

METHOD AUTOMATION AND ANALYSIS

We have created user friendly Perl scripts to automate and manage the results from an analysis using the methods outlined here. The first (nuc_loc.pl) takes in FASTA formatted protein sequences and sequentially runs each through all tools mentioned above, with the exception of ACCpro which is only run on proteins containing an NLS, outputting an integrated results file. A post processing script (nuc_loc_post.pl) then parses this file, applies user defined filters for GO terms, NLS exposure levels and NucPred scores and outputs a summary file denoting which proteins pass the applied filters. These scripts are freely available on our website (http://bioinformatics.childhealthresearch.org.au/software/nuc_loc/). The entire *T. gondii* proteome was run through the code outlined above. To create the final dataset for filtering we manually integrated the results from the SecretomeP analysis, the ToxoDB GO terms and the extended experimental list of potential secreted proteins detailed above.

The signal peptide (SignalP) and InterproScan predictions in this study could have been exported directly from ToxoDB, and indeed our predictions mirror those of this database as expected. However, these tools are key components in the pipeline we provide and their inclusion enables its use for the investigation of proteins from organisms that are not as well characterised as *T. gondii* and that do not have these exceptional resources available.

Filtering strategy - Figure outlines the steps involved in filtering our dataset to reveal the proteins most likely to target the host cell nucleus and have a role in the manipulation of the host cell epigenome. In step 1, we wanted to identify those proteins that are most likely to target to a eukaryotic cell nucleus by using either NucPred or Pre-

dictNLS to predict nuclear localisation. Used in concert improves the sensitivity and specificity over using these tools alone (Brameier et al. 2007). Step 2 narrows down the list of nuclear localised proteins to a list of epigenetic modifiers where any of their predicted InterPro domain GO terms or other annotated GO terms matched any GO terms in either of the GOLink terms lists. In Step 3, we filtered the list to comprise all proteins computationally predicted as being secreted as well as all those from secretory organelles or experimentally confirmed as being secreted. These proteins are suggestive of a role in the host epigenome while the rest not either predicted or experimentally shown as being secreted were deemed to have an endogenous role.

REFERENCES

Ashburner M, Ball CA, Blake JA, Botstein D, Butler H, Cherry JM, et al. Gene ontology: tool for the unification of biology. The gene ontology consortium. Nat Genet. 2000; 25(1): 25-9.

Bada M, Hunter L. Enrichment of OBO ontologies. J Biomed Inform. 2007; 40(3): 300-15.

Bendtsen JD, Jensen LJ, Blom N, Von Heijne G, Brunak S. Feature-based prediction of non-classical and leaderless protein secretion. Protein engineering, design & selection: PEDS. 2004; 17: 349-56.

Brameier M, Krings A, MacCallum RM. NucPred - Predicting nuclear localization of proteins. Bioinformatics. 2007; 23: 1159-60.

Carruthers VB, Moreno SN, Sibley LD. Ethanol and acetaldehyde elevate intracellular [Ca2+] and stimulate microneme discharge in *Toxoplasma gondii*. Biochem J. 1999; 342(Pt 2): 379-86.

Cheng J, Randall AZ, Sweredoski MJ, Baldi P. SCRATCH: a protein structure and structural feature prediction server. Nucleic Acids Res. 2005; 33: 72-6.

Chi SM, Nam D. WegoLoc: accurate prediction of protein subcellular localization using weighted Gene Ontology terms. Bioinformatics. 2012; 28: 1028-30.

Cokol M, Nair R, Rost B. Finding nuclear localization signals. EMBO Rep. 2000; 1: 411-5.

Dingwall C, Laskey RA. Nuclear targeting sequences - a consensus? Trends Biochem Sci. 1991; 16(12): 478-81.

Francis RW. GOLink: finding cooccurring terms across gene ontology namespaces. Int J Genomics. 2013; 2013: 10.

Gajria B, Bahl A, Brestelli J, Dommer J, Fischer S, et al. ToxoDB: an integrated *Toxoplasma gondii* database resource. Nucleic Acids Res. 2007; 36: D553-6.

Hunter S, Jones P, Mitchell A, Apweiler R, Attwood TK, Bateman A, et al. InterPro in 2011: new developments in the family and domain prediction database. Nucleic Acids Res. 2012; 40(Database issue): D306-12.

Mei S, Fei W, Zhou S. Gene ontology based transfer learning for protein subcellular localization. BMC Bioinformatics. 2011; 12: 44.

Mungall CJ. Obol: integrating language and meaning in bio-ontologies. Int J Genomic. 2004; 5(6-7): 509-20.

Nickel W. The unconventional secretory machinery of fibroblast growth factor 2. Traffic. 2011; 12: 799-805.

Pollastri G, Przybylski D, Rost B, Baldi P. Improving the prediction of protein secondary structure in three and eight classes using recurrent neural networks and profiles. Proteins. 2002; 47(2): 228-35.

Rubartelli A, Cozzolino F, Talio M, Sitia R. A novel secretory pathway for interleukin-1β, a protein lacking a signal sequence. EMBO J. 1990; 9: 1503-10.

Tinland B, Koukolíková-Nicola Z, Hall MN, Hohn B. The T-DNA-linked VirD2 protein contains two distinct functional nuclear localization signals. Proc Natl Acad Sci USA. 1992; 89(16): 7442-6.

Zdobnov EM, Apweiler R. InterProScan - an integration platform for the signature-recognition methods in InterPro. Bioinformatics. 2001; 17: 847-8.

Zhou XW, Kafsack BFC, Cole RN, Beckett P, Shen RF, et al. The opportunistic pathogen *Toxoplasma gondii* deploys a diverse legion of invasion and survival proteins. J Biol Chem. 2005; 280: 34233-44.
